# 35 Years of TFAM Research: Old Protein, New Puzzles

**DOI:** 10.3390/biology12060823

**Published:** 2023-06-06

**Authors:** Natalya Kozhukhar, Mikhail F. Alexeyev

**Affiliations:** Department of Physiology and Cell Biology, University of South Alabama, Mobile, AL 36688, USA

**Keywords:** mitochondria, mitochondrial transcription factor A (TFAM), mitochondrial DNA, mitochondrial DNA transcription, mitochondrial DNA replication, mitochondrial biogenesis, mitochondrial DNA repair

## Abstract

**Simple Summary:**

Transcription Factor A Mitochondrial (TFAM) was first purified 35 years ago as an activator of mitochondrial transcription. Because of its critical contributions to mitochondrial DNA transcription and replication, this protein was viewed as a master regulator of mitochondrial biogenesis. However, now it is clear that TFAM’s cellular role is more nuanced than previously thought. In this review, we attempted to compile and assess various, at times contradictory, lines of evidence supporting this protein’s diverse roles. It is carried out in the belief that the resolution of the existing contradictions is a prerequisite for further advancements in the field.

**Abstract:**

Transcription Factor A Mitochondrial (TFAM), through its contributions to mtDNA maintenance and expression, is essential for cellular bioenergetics and, therefore, for the very survival of cells. Thirty-five years of research on TFAM structure and function generated a considerable body of experimental evidence, some of which remains to be fully reconciled. Recent advancements allowed an unprecedented glimpse into the structure of TFAM complexed with promoter DNA and TFAM within the open promoter complexes. These novel insights, however, raise new questions about the function of this remarkable protein. In our review, we compile the available literature on TFAM structure and function and provide some critical analysis of the available data.

## 1. Introduction

Life, as we know it, has numerous manifestations, but no uniformly accepted definition. However, living systems must sustain and reproduce themselves. These processes are dependent on chemical energy, which living systems absorb from the environment, typically in the form of nutrients, and energy capture, most often in the form of ATP, which is casually referred to as the “universal energy currency” of life [1]. Therefore, ATP production is central to life as we know it. 

In animal systems, the most efficient extraction of energy from biological fuels occurs in the mitochondria, which are double-membrane-bound organelles that host many biochemical processes, ultimately leading to the production of reducing equivalents in the form of NADH and FADH_2_. These reducing equivalents are then “burnt” by the respiratory chain; the released energy is stored in the form of an electrochemical gradient across the mitochondrial inner membrane and captured in the high-energy bonds of ATP [2,3]. This process is so fundamental to eucaryotic organisms that, to date, only one eucaryotic genus that lacks mitochondria and all hallmark proteins responsible for mitochondrial function, *Monocercomonoides*, has been identified. These organisms also lack any other mitochondria-related organelles, such as hydrogenosomes or mitosomes [4].

Mitochondria are unique among metazoan organelles in that they are the only organelles other than the nucleus to store genetic information, which they do in the form of mitochondrial DNA (mtDNA). 

## 2. Mitochondrial DNA

mtDNA organization is surprisingly diverse across eucaryotes. Kolesnikov and Gerasimov [5] identified at least six types of mtDNA organization:

(1) Circular molecule 11–28 kbp.
(2) Circular molecule 22–1000 kbp.
(3) Circular molecule larger than 22 kbp accompanied by plasmid-like molecules.
(4) Heterogeneous population of circular molecules.
(5) Homogenous population of linear molecules.
(6) Population of heterogeneous linear molecules.

**Figure 1:**
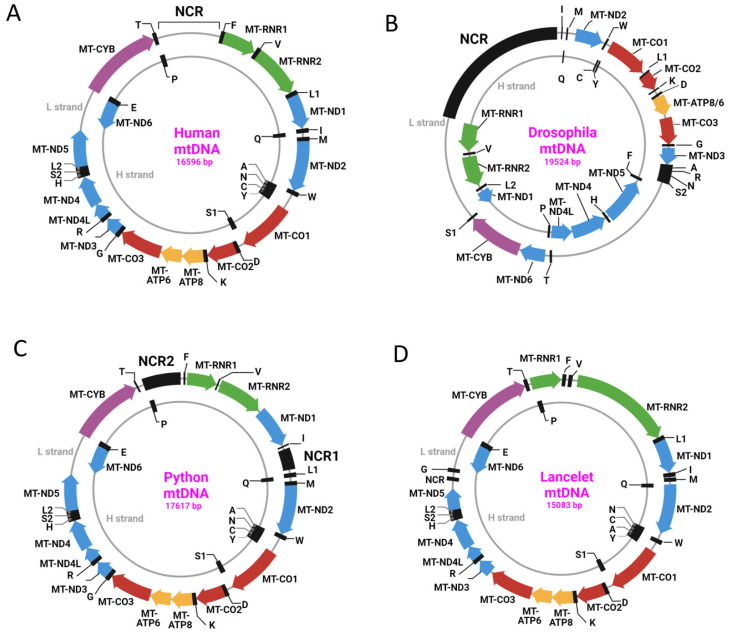
Examples of variability in mitochondrial genome organization among type 1 mitochondrial genomes. (**A**) h-mtDNA (GenBank NC_012920.1); (**B**) *Drosophila melanogaster* mtDNA (GenBank NC_024511.2). Note altered distribution of genes between L- and H-strands as compared to human mtDNA; (**C**) *Python bivittatus* mtDNA (GenBank NC_021479.1). Note the presence of two NCRs; (**D**) *Branchiostoma floridae* mtDNA (GenBank NC_000834.1). Note the severely diminished NCR (129 bp). tRNA genes are designated by black single-letter codes for the corresponding amino acids.

Even within types, mtDNA organization varies considerably. Thus, within type 1, which includes, among others, human mtDNA (h-mtDNA), there is considerable variation in gene order (Figure 1). 

h-mtDNA is the best-studied of all mtDNA molecules, and therefore, our view of mtDNA is somewhat anthropocentric. Accordingly, this review will focus primarily on human TFAM (h-TFAM) and its role in h-mtDNA metabolism.

Briefly, h-mtDNA and mouse mtDNA are double-stranded circular molecules that can be present in cells in several topological forms, including supercoiled monomeric circles, catenanes, oligomers, and complex multimeric networks [6,7]. The functional significance and prevalence of these topological forms remain largely obscured. However, their proportion is not insignificant. It has been reported that in different organs of mice, only 46–68% of all mtDNA molecules are monomeric circles [8].

Due to their differences in nucleotide composition, mtDNA strands can be separated in CsCl alkaline buoyant density gradients based on the differential ionization of their G and T residues in alkaline solutions [9,10,11,12]. The resulting heavy and light mtDNA strands (H-strand and L-strand, respectively) have different coding capacities, with most mitochondrial genes encoded on the L-strand [13,14]. This notion held true for 4200 of 4205 vertebrate mitochondrial genomes examined by Barroso Lima and Prosdocimi [15].

**Figure 2:**
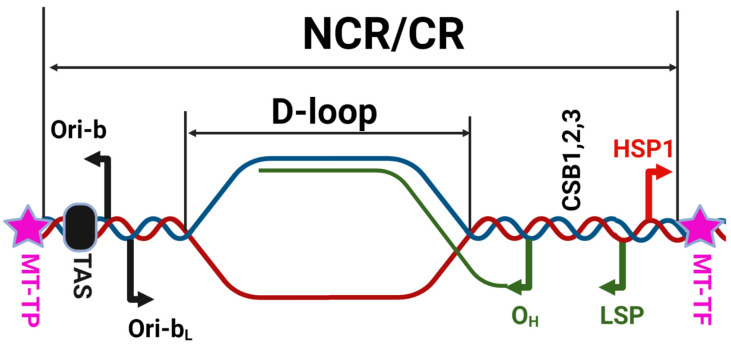
D-loop region of h-mtDNA and the relationship between the D-loop and the NCR/CR. CSB1, 2, and 3 are conserved sequence blocks regions 1, 2, and 3, respectively. O_H_, origin of the mtDNA H-strand replication, strand-coupled model [17,18,19]. TAS, replication-termination-associated sequence. Ori-b and Ori-b_L_, origins of the H- and L-strand mtDNA replication [20,21]. Bent green line, nascent H-strand. MT-TP and MT-TF, genes for mitochondrial tRNAs for proline and phenylalanine, respectively.

With the exception of the control region (CR; a.k.a. the noncoding region (NCR), 1122 bp located between genes encoding MT-TP and MT-TF), there are no major NCRs in h-mtDNA. The CR is believed to play a major role in mtDNA maintenance because it houses two of the three promoters for mitochondrial transcription, the termination-associated sequence (TAS), conserved sequence blocks (CSBs), and the origin of H-strand replication (Ori_H_, strand-asynchronous model) or origins for both H- and L-strand replication (Ori-b and Ori-b_L_, conventional strand-coupled Okazaki-fragment associating (COSCOFA) model [6]) (Figure 2). 

Remarkably, despite its attributed significance, the NCR may be severely reduced in some taxa (e.g., in lancelets), whereas in other taxa (e.g., in pythons), there may be two NCRs (Figure 1C,D). In addition, the NCR of various species, including primates, may contain repetitive elements that are absent from the human NCR [16]. This suggests that the NCR may play different roles in different taxa.

In some, but not all, h-mtDNA molecules, a so-called D-loop can be observed (Figure 2). This structure is thought to be a result of an abortive attempt at H-strand replication terminated at the TAS. It results in the formation 7S DNA, which forms a triple helix structure with two parental mtDNA strands. D-loop is variably defined as either 650 bp long [6] or ~1000 bp long [19]. The latter length definition resulted in the erroneously interchangeable use of the terms D-loop and NCR/CR, the obvious difference being that the D-loop is a physical structure that can be observed electron-microscopically only in some mtDNA molecules, whereas the NCR/CR is a conceptual structure present in all by definition.

## 3. TFAM

The maintenance, expression, and organization of mtDNA depend on nuclear DNA-encoded TFAM. This protein is a member of the HMGB subfamily of the high-mobility group (HMG) proteins and plays a prominent role in mitochondrial and cellular physiology through its critical contributions to mtDNA transcription and replication. Both nuclear and mitochondrial localizations of TFAM have been reported [22,23,24]. However, TFAM knockout (KO) cells have been reported recently, indicating that neither nuclear nor mitochondrial TFAM is essential for cell viability [25].

Whole-body TFAM KO in mice is embryonically lethal and accompanied by severe mtDNA depletion [26]. However, variable phenotypes were observed in mouse tissue-specific TFAM KOs, some relatively mild [27,28,29]. Thus, mice with TFAM KO in the basal layer of the epidermis, which contains stem cells responsible for its renewal, resulted in mtDNA depletion and a loss of respiratory complexes. However, epidermal development and skin barrier function were not impaired [27]. Similarly, the heart relies heavily on mitochondrial respiration to produce ATP for constant contractions. Therefore, 37% of the cardiac myocyte volume is occupied by mitochondria [30]. Still, mice with TFAM KO in the heart and skeletal muscle (including the diaphragm) survived for up to 150 days [31]. Considering that TFAM ablation in cultured cells is accompanied by mtDNA loss and the inability to produce ATP through oxidative phosphorylation [25], these phenotypes have no simple explanation (see section Limitations of the available experimental systems). 

## 4. TFAM Domain Organization

**Figure 3:**
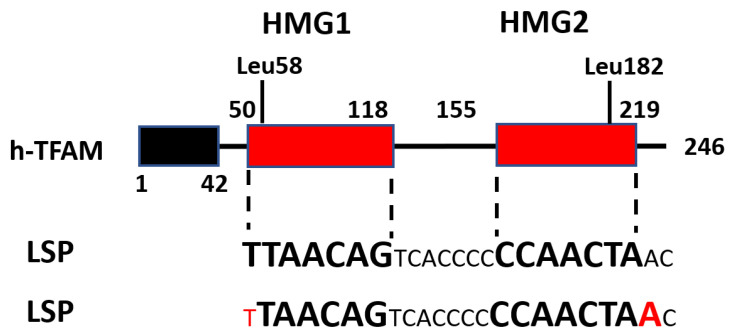
h-TFAM organization and interaction with the human light strand promoter (hLSP). Top: The domain structure of TFAM as per UniProt [41]. Middle and bottom: hLSP residues contacted by h-TFAM HMG1 and HMG2 domains, as per Hillen et al. [42] and Rubio-Cosials et al. [33], respectively. Residues contacted by HMG1 and HMG2 are in large bold font. Note that in the Rubio-Cosials et al. structure, TFAM binding is shifted by one nucleotide away from the transcription start site (TSS; red font). Further, there is a one-base difference in the number of nucleotides contacted by HMG1 and HMG2.

Proteins of the HMGB family are characterized by the presence of two HMG-B boxes. Accordingly, TFAM also has two HMGB boxes, discordantly named in the literature as either HMG1 and HMG2 [22,32,33,34,35], or HMG-boxA and HMG-boxB [36,37], or HMG box1 and HMG box2 [22,38,39,40] (Figure 3). These two boxes split mature proteins into five domains: leader, HMG1, linker, HMG2, and tail. Unlike nuclear HMGB proteins, TFAM possesses a sixth domain, the Matrix Targeting Sequence (MTS), a 42-amino-acid (aa) presequence that is removed from human and murine proteins upon mitochondrial import and is not present in the mature protein (Figure 3). 

The literature is discordant with respect to the number, identity, and boundaries of TFAM domains. We counted eight different annotations of TFAM domains in various studies (Table 1). This discordance can create ambiguity when attempting to define the functional importance of a given domain (e.g., [43]). 

## 5. Functional Roles of TFAM Domains

*MTS* TFAM MTSs are not conserved evolutionarily; however, most are at least partially functional in human cells, including MTSs of TFAM orthologs (oTFAMs) from yeast, *Caenorhabditis elegans*, *Drosophila melanogaster*, and even the extremely short 11-aa MTS from Australian ghost shark *Callorhinchus milii* TFAM [25]. Functionally, the MTS interacts with mitochondrial protein import machinery to translocate TFAM across mitochondrial outer and inner membranes into the mitochondrial matrix.

*Leader sequence*. The leader sequence is short, without notable conservation, and of variable length (7 aa in humans, 6 aa in mice). It is likely TFAMs with short aa sequences preceding HMG1 (e.g., 11 aa in *C. milii*) have no or very shortened leader sequences. However, precise delineation of the length of the leader sequences awaits the experimental determination of MTS cleavage sites in oTFAM. The leader sequences are the least conserved of all TFAM domains. Therefore, in our opinion, their incorporation into the HMG1, which is a conserved sequence domain, is unwarranted. In fact, neither the original Parisi paper [45] nor homology-based annotations [41,46,47] incorporate the leader sequence into HMG1.

*HMG1* (a.k.a. HMG box1, a.k.a. HMG-boxA). In different annotations (Table 1), this domain may have a length of 66–83 aa. In vitro, isolated HMG1 has DNA-binding properties, whereas the second HMG box (HMG2, a.k.a. HMG box2 and HMG-boxB) is unable to bind DNA on its own [32,37]. Based on glycerol gradient sedimentation analysis, TFAM in solution is monomeric [48]. Similarly, based on sedimentation velocity analysis, TFAM exists as a monomer in solution, but dimerizes upon DNA binding [37]. Alternatively, size-exclusion chromatography analysis indicated that TFAM in solution exists as a dimer [49] or, based on the combined data from size-exclusion chromatography, analytical ultracentrifugation, and nuclear magnetic resonance studies, that monomeric and dimeric forms of TFAM exist in equilibrium [32]. It has been suggested that either HMG2 [49] or the C-terminal tail [32] is responsible for TFAM dimerization. However, the structure of the h-TFAM-DNA complex indicates that the dimer interface may lie within HMG1 [50].

*Linker*. This domain is 27–39 aa long (Table 1). The linker contains a number of positively charged residues. It is thought that these residues help to compensate for repulsion between sugar-phosphate backbone phosphates, which are brought closer by TFAM-mediated DNA bending [33]. Of all the TFAM domains, the linker is the most sensitive to structural variations in DNA [51]. The reversible unfolding of the linker resolves the tension created by the sharp bending of DNA, which naturally tends to assume a linear conformation [51]. The linker passes perpendicularly to the DNA strand and connects HMG1 and HMG2 positioned at either side of the double helix. In doing so, the linker contributes to an overall U-bend shape of DNA. Accordingly, the L6 TFAM mutant, in which six positively charged residues in the linker (Lys136, His137, Lys139, Arg140, Lys146, and Lys147) are replaced with alanine, has a severe DNA bending defect [36]. The linker was also shown to increase the affinity of the isolated HMG2 domain to DNA [32]. In the free protein, the linker is disordered. However, upon DNA binding, it assumes an α-helical conformation [32,33,36,37,50,52].

*HMG2.* This domain is 65–80 aa long (Table 1). HMG2 is positioned on the opposite side of the DNA double helix from HMG1. As stated above, isolated HMG2 is unable to bind DNA in vitro [32,37]. This fact, along with the lack of identifiable DNA-intercalating residues, led to the notion that HMG2 is a noncanonical HMG domain [32]. This notion was subsequently challenged by crystallographic studies (see below) [33,36,50]. It has been recently suggested that HMG2 may play a leading role in determining TFAM species specificity [25]. 

*Tail*. This domain is 12–27 aa long (Table 1). Typically, the C-terminal tails of HMGB proteins are negatively charged [53] However, the tails of most TFAMs have a net positive charge. Curiously, the tails of oTFAM from cat, mouse, pig, and pika are negatively charged, yet these proteins support the replication of h-mtDNA [25]. Moreover, h-TFAM can substitute for murine TFAM in transgenic animals [54]. 

Collectively, these observations suggest that TFAM’s function does not critically depend on the charge of its C-terminus. This is in contrast to the findings of an earlier study, which suggested the importance of the negative charge of the TFAM tail for transcription from the LSP [34]. 

In organello studies using isolated mitochondria demonstrated that the import of full-length TFAM into isolated mitochondria stimulates transcription, whereas the import of tailless TFAM does not [55]. Therefore, the TFAM tail may be important for transcription.

Removal of the tail results in 1034-, 825-, and 653-fold decreases in the affinity of TFAM for the LSP, TAS, and nonspecific (NSP) DNA, respectively [56]. Surprisingly, recent indirect evidence suggests that loss of the C-terminal tail may either increase the affinity of TFAM for DNA rather than decrease it [57] or have no effect [58]. Since the loss of the tail affects TFAM’s affinity for LSP the most, these observations suggest that the tail may be involved in promoter recognition (sequence-specific DNA binding [37]) and nucleoid organization [56]. However, another study revealed that deletion of the tail decreases TFAM affinity to LSP only approximately twofold, and that tail-less TFAM binds NSP DNA with near wild-type affinity [37]. Yet another study did not reveal any sizable reduction in TFAM’s affinity to HSP1 upon the removal of the 25 C-terminal aa [58]. 

The role of the TFAM tail in mtDNA replication remains incompletely defined. A seminal study by Matsushima et al. established that chicken TFAM retaining only the three tail aa most proximal to HMG2 retains full activity in mtDNA replication in TFAM-haploinsufficient DT40 cells. As a caveat, that study reported that the TFAM tail contains both stimulatory and inhibitory sequences for mtDNA replication [22]. Similarly, tailless TFAM rescues the mtDNA copy number (mtCN) in TFAM-depleted HeLa cells, indicating that the TFAM tail may be dispensable for mtDNA replication [59,60]. However, the conclusions in these studies were drawn by examining cells in which both wild-type and tailless TFAMs were co-expressed. Therefore, the contribution of complex interactions between these two forms could not be excluded.

It has been shown that the last 10 aa of the TFAM tail are dispensable for transcription from the LSP promoter in vitro. However, the deletion of 15 or more residues severely impairs transcription [34,35,60]. Conversely, transplantation of the 29-aa tail of h-TFAM onto its transcriptionally inactive yeast ortholog Abf2 converts it into a transcriptionally active chimeric protein [34]. Additionally, the h-TFAM tail has been suggested to interact with and recruit another mitochondrial transcription factor, TFB2M [61]. However, contradictory evidence indicates that the TFAM tail crosslinks with POLRMT, but not TFB2M [38].

Together, these observations support the critical role played by the TFAM tail in mitochondrial transcription. However, recent in vitro studies employing a more physiological template that included both the H-strand promoter 1 (HSP1) and LSP, as well as the region between these promoters, painted a different picture: the tailless TFAM supports transcription from LSP, but is completely unable to activate transcription from HSP1 [54].

Recently, it has been demonstrated that the TFAM tail is dispensable for mtDNA replication and transcription in situ. This evidence represents a challenge to the current model of mtDNA transcription, which postulates a critical role for the TFAM tail in the assembly of the mitochondrial transcription apparatus [43].

## 6. TFAM DNA Binding

**Figure 4:**
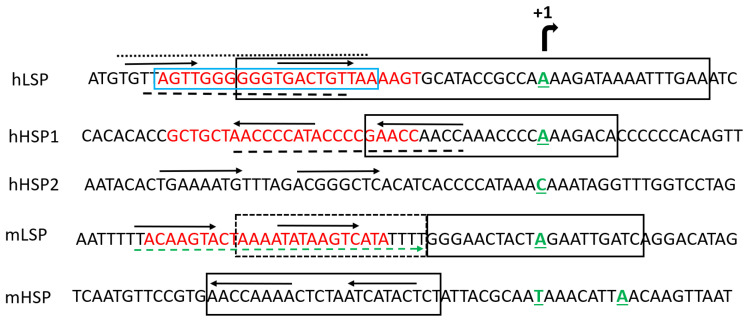
TFAM footprinting of mitochondrial promoters in mouse and human cells. The nontemplate strand is shown. TSSs are aligned and designated by the bent arrow at the top. The +1 nucleotides of transcripts are green, bold, and underlined (human and mouse HSP1 and LSP initiating nucleotides are as per [63] and [64], respectively; hHSP2 is as per [65]). Promoter sequences are enclosed in solid black boxes (hHSP1 and hLSP as per [63], mLSP and mHSP as per [66]). Horizontal arrows, putative HMG binding sequences, and their direction as per [67]. Blue box, TFAM-interacting LSP region in [42]. Broken box, sequences important for the preinitiation complex formation at mLSP, as per [66]. Red font, TFAM footprints as per [62]. Broken lines underline TFAM footprints as per [68]. Dotted line over the sequence, TFAM footprint as per [69]. The broken green arrow under the sequence indicates TFAM footprint as per [70].

Mammalian TFAMs bind mitochondrial DNA (mtDNA), both sequence-specifically and nonspecifically. In mammalian cells, sequence-specific binding occurs upstream of the LSP and mitochondrial HSP1 and H-strand promoter 2 (HSP2), producing specific DNase I footprints [34,62]. These footprints are in reasonable agreement among studies (Figure 4). The sequential cooperative binding model suggests that HMG1 binds DNA first. This binding then facilitates the folding of the unstructured linker into a 29-aa α-helix, bringing the HMG domains closer and facilitating DNA binding by HMG2 [47].

Abundant evidence indicates that at HSP1, TFAM binds in the reverse orientation compared with LSP in simple in vitro systems containing just promoter DNA and TFAM [34,50,62]. Remarkably, this orientation is reversed in crystals that also contain POLRMT and TFB2M [42]. The fact that the orientation of TFAM binding upstream of mitochondrial promoters might not be an intrinsic property of the TFAM/DNA sequence pair, but could be reversed by accessory proteins and/or DNA melting, highlights the necessity for caution when making inferences from the orientation of TFAM binding.

Crystal structures show that TFAM binding at LSP, HSP1, or NSP DNA induces sharp (~180°) bends, thus facilitating mtDNA compaction and assembly into nucleoids [33,36,44,50], the structures in which mtDNA replication and transcription are thought to occur. It has been suggested that TFAM bending at LSP positions the TFAM tail near the TSS and, therefore, is necessary for the full activation of transcription. This notion is consistent with the observation that TFAM mutants defective in DNA bending are defective in initiating transcription at the LSP in vitro, but fully active at HSP1 transcription [36,50], where TFAM binds in the opposite orientation and, therefore, is in the vicinity of the TSS, regardless of DNA bending [36]. However, recent evidence from the higher-order structures of the transcriptional apparatus indicates that TFAM binds in the same orientation at the LSP and HSP1 [42]. Therefore, it is unclear why TFAM bending mutants have a greater effect on LSP transcription than on HSP1 transcription in vitro [36,50], or why the effects of bending mutations on mitochondrial promoters are reversed in situ [43].

Estimates of the size of the TFAM footprint on DNA vary among studies almost fourfold, falling in the range 10–37.2 bp [34,62,68,69,70,71,72,73,74]. The identities of TFAM-protected nucleotide sequences upstream of mitochondrial promoters also vary slightly among studies (Figure 4). In many studies, TFAM shows the highest affinity for the LSP, followed by HSP1 and NSP. However, some available evidence suggests that there may be little or no difference in TFAM binding affinities at specific sites upstream of mitochondrial promoters and at NSP regions of mtDNA, at least under certain experimental conditions [32,49,52,75]. Additionally, estimates for TFAM affinity to LSP vary ~40-fold among studies [32,49].

Most mitochondrial TFAM is believed to exist in the mtDNA-bound state [76,77]. Dissociation of TFAM from mtDNA is believed to result in its destabilization and rapid degradation by the mitochondrial AAA+ Lon protease [40,78]. However, more recent evidence indicates that TFAM levels may remain unchanged (and, therefore, TFAM may be protected from Lon protease degradation) in cells severely depleted of mtDNA [79]. The mechanism for this resistance to degradation remains obscure, despite its importance for potential therapeutic manipulation of mitochondrial metabolism through modulation of TFAM levels.

## 7. TFAM Residues Interacting with mtDNA

In 2011, two groups reported crystal structures of h-TFAM complexed with DNA oligonucleotides encompassing the LSP [33,36]. These were the first structures of a native HMGB protein complexed with DNA [36]. Each group employed a subtly different strategy for crystallization. Rubio-Cosials et al. used a 22-bp oligonucleotide sequence TAACAGTCACCCCCCAACTAAC fully protected in footprinting assays [62,69] and a full-length mature h-TFAM (residues 43–246) with N-terminal addition of 2 aa: MG, and C-terminal addition of 8 aa: LQHHHHHH [33]. In contrast, Ngo et al. used a longer, 28-bp promoter oligo TGTTAGTTGGGGGGTGACTGTTAAAAGT and a full-length mature h-TFAM (residues 43–246) with a long 42-aa N-terminal extension MSEGSSHHHHHHSSGLVPRGSHMSEASMSETGGQQMSEGRGS and a native C-terminus [36].

Overall, the structures were similar. Both groups identified the hallmark feature of DNA complexed with TFAM: a sharp 180° bend. Neither structure resolved the last nine TFAM residues due to crystallographic disorder, presumably because of intrinsic flexibility [33]. Both groups identified L58 as an intercalating residue in HMG1. Both groups pointed out that, in their structures, HMG2 no longer behaves as a noncanonical HMG domain and does indeed insert a residue into the DNA minor groove. Strikingly, though, this residue was L182 in the Rubio-Cosials et al. structure, whereas Ngo et al. identified the intercalating residue as P178 [33,36]. In a follow-up study, Ngo et al. corrected P178 to L182 (ref. [50], Figure 1c) and added that N163 and P178 also contribute to a DNA-bending wedge [50].

Another notable discrepancy between published TFAM/DNA structures is in the identity of TFAM residues making DNA contacts (Table 2). While it is plausible that the discordance in residues contacting DNA between LSP, HSP1, and NSP DNA is explainable by differences in the nucleotide sequences co-crystallized with TFAM, it is less clear why, e.g., V166, which is a part of the HMG2 hydrophobic core, makes contact with LSP DNA in [33], but not in [36]. Alternatively, it is unclear why K69 makes DNA contact in TFAM-LSP crystals by Rubio-Cosials et al. [33] and in TFAM-NSP DNA crystals by Ngo et al. [50], but not in TFAM-LSP crystals by the same authors [36]. It is possible, though, that some of this apparent discrepancy is due to intrinsic flexibility and mutually induced fitting of TFAM and DNA proposed earlier [33]. However, it would be helpful to exclude the possible contribution of variability in non-native N- and C-terminal appendages and truncations in TFAM variants that were used for crystallization in these studies [33,36,50].

As noted above, TFAM binds mtDNA in a minor groove [33,36]. This type of binding is uncommon among proteins recognizing specific DNA sequences [80,81], although sequence-specific recognition through the DNA minor groove has been described [82,83]. HMG proteins form hydrogen bonds in the minor groove. To achieve specificity, they rely on recognition of the DNA shape and flexibility [83]. Therefore, it is important to understand the exact mechanism of sequence recognition and discrimination of NSP sequences by TFAM. 

## 8. TFAM and mtDNA Compaction

Mammalian mtDNA molecules have a contour length of ~5 ϻm and, therefore, require compaction to fit within nucleoids, which are mtDNA-containing submitochondrial structures. Nucleoids were first observed microscopically in cells stained with fluorescent DNA intercalator DAPI [84]. They can be spherical with a diameter of ~70 nm [74,85] or ellipsoids with dimensions 35 × 45 × 75 nm [86]. Their analysis revealed a complex protein composition [77,87,88,89,90,91,92], and TFAM was suggested to be the main constituent of nucleoids in mammals [77]. However, in a number of studies, heterogeneity in nucleoid TFAM content is evident, and nucleoids with little or no TFAM have been observed [75,89,93,94,95]. The mechanistic basis behind this uneven TFAM distribution between nucleoids within a single cell and its biological significance remain to be elucidated.

The idea that TFAM contributes to mtDNA packaging was initially derived from observations that TFAM induces negative supercoiling in relaxed plasmids [56,96]. Super-resolution microscopy studies of cultured cells suggested that there could be as few as 1 mtDNA and as many as 1000 TFAM molecules per nucleoid [74]. At this ratio, there is enough TFAM to “completely coat” mtDNA. There are, however, conflicting estimates, some suggesting that there could be as few as 50 TFAM molecules per mtDNA, which is insufficient for complete coating [48,97,98]. At what is considered by many near-physiological TFAM/DNA ratios (1:20 bp), a range of structures can be observed in vitro, from nearly naked DNA molecules to fully compacted nucleoprotein complexes, in which the DNA contour length is reduced >14-fold [99]. Since highly compacted nucleoids may present challenges to mtDNA transcription and replication [99], it appears that at physiological TFAM/DNA ratios, a range of nucleoids would be present, ranging from fully compact, transcription/replication incompetent to “loose,” in which transcription and replication of mtDNA can occur. Indeed, in vivo data show that overexpression of TFAM in mice can suppress mtDNA transcription; however, mtDNA replication remains increased or unaffected [100]. 

**Figure 5:**
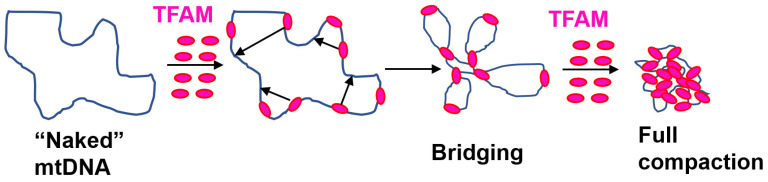
A schematic of mtDNA compaction through DNA bending and cross-strand bridging. Increasing the DNA TFAM/DNA ratio leads to increased DNA compaction.

Cooperative binding of TFAM to DNA leads to dramatic reversible changes in the extent of mtDNA compaction upon relatively minor changes in TFAM abundance. Compaction has been suggested to be driven by DNA bending [36,50,52,101] and by TFAM-mediated DNA cross-strand bridging [49,102] (Figure 5). A dissenting view rules out mtDNA compaction by TFAM-mediated wrapping and DNA looping/bridging based on biophysical studies [101]. It also holds that TFAM-mediated DNA looping observed with atomic force microscopy could be an artifact and advocates a “flexible hinge” model, in which the NSP DNA binding of TFAM increases the intrinsic flexibility of the DNA, resulting in DNA being bent at a variety of angles. This model can yield an apparent ‘rigid’ bending angle, which would be an average of all the possible angles that can be formed on TFAM binding [101].

Importantly, at what is considered by many to be a physiological TFAM/DNA ratio (1: 15 bp), the resulting nucleoids are much larger than those observed in situ (~300 nm vs. ~70 nm), and full compaction is only achieved at ratios exceeding 1 TFAM per 6 bp [102]. This difference is difficult to explain by the 25% longer DNA molecule used for compaction or by nucleoid flattening by rotary shadowing. Therefore, it cannot be excluded that some other factors may cooperate with TFAM in mtDNA compaction.

The latest model of nucleoid formation postulates the leading role of TFAM-driven phase separation, which critically depends on the TFAM C-terminus [44]. Further, the properties of in vitro-generated condensates containing TFAM and mtDNA closely resemble those of mitochondrial nucleoids [57]. Therefore, it would be interesting to examine nucleoids in cells that express truncated TFAM.

## 9. TFAM and Mitochondrial Transcription

TFAM was originally purified as a transcriptional activator in 1988 [48]. However, to this day, the role of TFAM in mitochondrial transcription remains incompletely defined. In vitro, low-level transcription from mitochondrial promoters has been observed in the absence of TFAM, suggesting that the core mitochondrial transcription apparatus might be a regulated two-component system consisting of POLRMT and TFB2M and regulated by TFAM [62,103,104,105,106,107]. 

**Figure 6:**
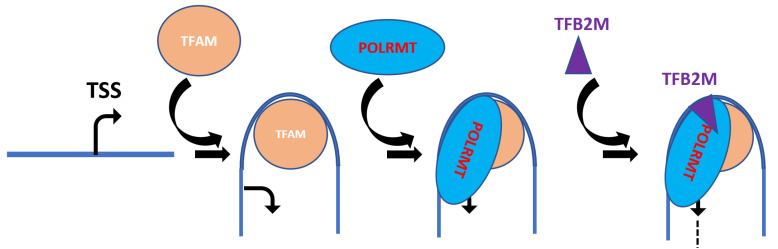
The sequential model for mitochondrial transcription initiation. (1) TFAM binds to a high-affinity binding site upstream of a mitochondrial promoter (designated by the bent arrow at the TSS) and induces a sharp bend in mtDNA; (2) POLRMT is recruited to the mtDNA/TFAM complex, presumably through interaction with TFAM C-terminal tail; (3) Recruitment of TFB2M to the mtDNA/TFAM/POLRMT ternary complex facilitates promoter melting, recruitment of the initiating nucleotide, and initiation of RNA synthesis (broken black line).

However, more recent evidence indicates that in the absence of TFAM, mtDNA is lost, and therefore, no transcription can occur [25,26]. This maxim notwithstanding, nucleoids with little or no TFAM staining have been observed in many studies [75,89,93,94,95], thus leaving open the possibility that conditions for TFAM-independent transcription could be available in vivo. The sequential model of mitochondrial transcription (Figure 6) derived from in vitro and structural studies postulates that the first step in transcription initiation is TFAM binding upstream of mitochondrial promoters (region −36 to −17 nucleotides upstream of the TSS) [42,108,109]. 

Alternatively, it has been stated that for optimal transcription initiation, TFAM has to bind exactly 10 bp (one helical turn) upstream of the TSS [67]. This initial TFAM binding induces DNA bending and the recruitment of POLRMT to the TSS, presumably through specific interactions between the TFAM HMG2 domain and the long ‘tether’ helix in the N-terminal domain of POLRMT [42,109]. Previously, it was suggested that TFAM might mediate the assembly of transcription complexes through interactions of its C-terminal tail with TFB2M [61]. Recent in situ data indicate that the TFAM tail is dispensable for mitochondrial transcription, suggesting that it is unlikely that the TFAM tail plays a critical role in the assembly of the mitochondrial transcription apparatus [43]. Therefore, it appears to be more likely that TFAM HMG2–POLRMT interactions drive the assembly of transcription complexes [42]. The preinitiation complex of TFAM and POLRMT is transcription-incompetent and requires TFB2M to melt the promoter and initiate transcription [35,42]. Overall, three proteins are required for mitochondrial promoter-dependent transcription initiation in human cells: TFAM, POLRMT, and TFB2M.

The current model of mitochondrial transcription is congruent with most structural and in vitro data; however, some unresolved issues remain. It remains unclear how specific transcription is initiated in heterologous systems. For example, it has been claimed that h-TFAM does not footprint the murine LSP [110]. Still, mice with heart-specific expression of h-TFAM are viable and healthy at the age of 52 weeks, with near-normal steady-state levels of mitochondrial transcripts [111]. Together, these observations suggest that sequence-specific TFAM binding might be optional for transcription initiation. In a similar vein, many heterologous TFAMs support the replication of h-mtDNA, which is dependent on LSP transcription [25], and yet, mtDNA sequences upstream of LSPs in these organisms appear too divergent to afford sequence-specific binding of corresponding TFAMs to human LSP. Alternatively, determinants of the sequence specificity of TFAM binding upstream of the LSP have to be very loose. 

The sequential model of mitochondrial transcription initiation posits that the initiating event in the assembly of transcription complexes is TFAM binding upstream of mitochondrial promoters [14,35,38,108]. Structural studies indicate that TFAM binding to HSP1 promoter DNA alone [50] is opposite to that found in open transcription complexes at the same promoter [42]. Therefore, it is unclear why and how TFAM flips its orientation at HSP1 upon recruitment of the POLRMT and TFB2M and DNA melting.

At some priming sites for mtDNA synthesis, such as the origin of the light strand replication (Ori_L_), transcription appears to be independent of TFAM [112,113]. In several studies, TFAM-independent transcription of mitochondrial promoters has been observed [62,103,104,105,106,107]. Furthermore, at HSP2, TFAM binding sites were reported both upstream (−15 to −10) and downstream (+1 to +5) of the TSS [106]. All these data are incongruent with the current model of mitochondrial transcription, thus limiting its general applicability. Note that, despite the evidence supporting the existence of HSP2 coming from the identification of transcripts with distinct 5’ ends corresponding to this promoter [65,114,115], reconstitution of HSP2 transcription in vitro [105,106], and proof from recent RNAseq studies [116], the existence of HSP2 is not universally accepted, primarily due to this promoter’s weakness and unusual location [117]. However, TFAM C-terminal truncations and mutations differentially affect the steady-state levels of HSP1 and HSP2 transcripts [25,43,118]. Collectively, these observations lend further credence to the existence of two HSPs and mechanistically distinct contributions of TFAM to the activities of these two promoters. 

Remarkably, in some organisms, oTFAMs are dispensable for mitochondrial transcription. For example, Abf2 (yeast oTFAM) is capable of DNA binding and bending, but plays no direct role in transcription [119].

## 10. TFAM and mtDNA Replication

TFAM’s role in mtDNA replication remains incompletely defined. It is well established that TFAM is essential in vivo, and whole-body TFAM KO is embryonically lethal [26]. This lethality is associated with severe mtDNA depletion [26]. Similarly, in cultured cells, TFAM KO leads to the loss of mtDNA [25]. However, in some tissue-specific TFAM KOs, both mtDNA and mitochondrial transcription were retained, although at reduced levels, for extended periods of time (see Section 14). In one study, TFAM KO in the heart and skeletal muscle of mice resulted in delayed death at 2−4 weeks of age [120]. In another study, similar mice survived for 12 weeks [28]. TFAM protein and mitochondrial transcript levels in the heart and skeletal muscle were reduced, as were the activities of respiratory complexes I and IV in the heart, but, surprisingly, not in the skeletal muscle [120]. Surprisingly, mtCN increased in the hearts of these animals between 2 and 4 weeks of age and remained steady up to 8 weeks of age [28]. In flies, TFAM overexpression had no effect on mtCN [121]. In differentiating mouse myoblasts, mtCN was decreased, despite a fourfold increase in TFAM expression [122]. In cultured cells, treatment with ethidium bromide reduced both TFAM expression and mtCN. However, upon drug withdrawal, mtCN recovered faster than TFAM levels, suggesting the optionality of TFAM for mtDNA replication [123]. Conversely, mtCN was unaffected by transient TFAM overexpression in cultured cells [98]. In POLRMT-depleted cells, mtDNA levels were reduced, despite unaltered TFAM levels [79]. Dramatically, in one study, mtCN levels were the highest in the tissue with the lowest TFAM expression [124]. However, other studies identified a positive correlation between TFAM expression and mtCN, at least at modest levels of TFAM overexpression and in TFAM haploinsufficiency [22,26,60,71,100,110].

The oldest and best-understood strand-displacement model of mtDNA replication posits that abortive L-strand transcripts prime mtDNA H-strand replication [17,18,19]. A fraction of LSP transcripts terminate at CSB2 and prime H-strand synthesis. Mitochondrial transcription elongation factor (TEFM) serves as an antiterminator and promotes the synthesis of near-genomic-length mitochondrial transcripts. However, evidence from TEFM KO experiments and RNAseq studies indicates that CSB3, rather than CSB2, is the main transcription attenuation site [116,125]. The primer generated at the LSP is extended by mitochondrial replisome and displaces the H-strand over ~70% of mtDNA length. Then, as soon as H-strand replication exposes the origin of the L-strand replication (O_L_), the synthesis of a new L-strand is initiated in the opposite direction [126]. In this model, TFAM is critically important for priming the replication of the mtDNA H-strand at O_H_. Curiously, initiation of mtDNA replication at O_L_ is apparently TFAM-independent, since POLRMT can prime L-strand synthesis in the absence of TFAM [112,113]. TFAM contributions to priming mtDNA replication in other models remain to be elucidated.

Overall, TFAM sequence space is remarkably flexible for mtDNA replication. Many oTFAMs can substitute for h-TFAM, and 730 aa substitutions that are conditionally permissive for h-mtDNA replication were reported in 204-aa mature h-TFAM after a survey of 29 oTFAMs [25]. Clearly, more similar mutations may be identified by examining more oTFAMs. In oTFAMs that support h-mtDNA replication, the HMG2 domain is the most conserved (the fewest conditionally permissive substitutions per aa and the most invariant aa), suggesting that it plays a leading role in TFAM species-specificity (the ability to support replication of the cognate mtDNA) [25]. Finally, the ability to support h-mtDNA replication could be imparted to chicken TFAM by “humanizing” as few as 8 of the 22 aa that are predicted to make contact with mtDNA, whereas this ability was not restored by “humanizing” 78 aa that do not make such contact [118]. This observation clearly indicates that TFAM residues that make DNA contacts play a leading role in TFAM’s ability to support replication of mtDNA.

In conclusion, while TFAM’s role in mammalian mtDNA replication has begun to emerge, it remains relatively underexplored in other taxa, and oTFAMs could be dispensable for mtDNA replication in other organisms, e.g., yeast [127,128,129].

## 11. TFAM and mtDNA Repair

The major DNA repair pathway in mitochondria is the Base Excision Repair (BER) pathway [130]. Within this pathway, monofunctional glycosylases may recognize and remove a damaged base leaving behind an abasic (AP) site, which requires further processing by short-patch BER. This processing includes AP incision with abundant AP endonuclease (APE1) ‘5 to the lesion and the removal of the resulting 5’ deoxyribosephosphate (dRP) group by DNA polymerase γ [130]. Recent data suggest that TFAM stimulates the incision of AP sites in mtDNA, reducing their half-life by 2–3 orders of magnitude to minutes, thus promoting their repair [131,132,133,134,135]. Earlier studies revealed preferential TFAM binding to DNA containing oxidatively damaged bases [136] and to DNA 4-way junctions [137], but surprisingly, not AP sites [136]. Moreover, TFAM was shown to inhibit BER enzymes 8-oxoguanine DNA glycosylase (OGG1), uracil-DNA glycosylase (UDG), APE1, and DNA polymerase γ [136]. In vivo, AP sites can also be incised by redundant activities of APE1 and bifunctional DNA glycosylases. Therefore, future studies should determine TFAM’s relative contribution to AP site processing vs. these redundant activities.

## 12. TFAM and Mitochondrial Biogenesis

TFAM has been called a “master regulator of mitochondrial biogenesis” [138]. Indeed, available evidence suggests that, at least in some experimental systems, such constituents of mitochondrial biogenesis as mtCN, mtDNA transcription, and translation of mtDNA-encoded polypeptides, as well as some mitochondrial functions, may closely parallel TFAM expression [22,60,98,110,139]. It was also reported that in some [22,74,77,110,140], but not all [48,97,98], experimental systems, endogenous TFAM levels are sufficient to completely cover mtDNA, assuming that in vivo TFAM footprints on mtDNA are of the same size as they are in vitro (23–30 bp [48,101]). 

That evidence notwithstanding, the relationship between TFAM expression and mitochondrial biogenesis is the subject of ongoing debate. Even in early studies, it was noted that TFAM haploinsufficiency resulted in an ~34% reduction in mtDNA mtCN and a decrease in mitochondrial transcript levels and protein expression in some tissues, but not in others [26]. This suggested a poor correlation between TFAM expression and the bona fides of mitochondrial biogenesis. TFAM overexpression may or may not result in an increased mtCN, depending on the tissue and expression level [100]. In contrast, induced TFAM overexpression reduced mtCN in cultured cells by 40–60% [141]. 

TFAM overexpression has either no effect or a detrimental effect on mitochondrial gene expression, depending on the tissue [100]. Examination of clones stably overexpressing either full-length h-TFAM or C-terminally truncated h-TFAM suggested that overexpression of the full-length TFAM does not inhibit transcription initiation, but inhibits elongation. In contrast, overexpression of the C-terminally truncated TFAM inhibits transcription initiation, but not elongation [56].

Mice expressing TFAM at a very high level developed OXPHOS deficiency and died soon after birth [100]. Changes in the TFAM expression level and mtCN had an opposite directionality in some tissues in a MERRF patient [124]. TFAM knockdown and overexpression in several cancer cell lines revealed that the relationships between TFAM knockdown or overexpression and mitochondrial biogenesis did not follow any particular pattern [142].

TFAM contributions to mtDNA replication are also controversial. We already mentioned that the whole-body TFAM KO is embryonically lethal [26,110], and TFAM KO in cultured cells results in the loss of mtDNA [25]. However, animals with TFAM KO in the heart survived for 12 weeks [120], which is remarkable considering the heart’s high reliance on mitochondrial function. Surprisingly, despite the presumed lack of TFAM, mtCN in the hearts of these animals increased between two and four weeks of age and then remained steady up to eight weeks of age [28]. In flies, TFAM overexpression did not alter mtCN [121]. Similarly, transient TFAM overexpression in cultured cells did not affect mtCN [98]. In ethidium bromide-treated cultured cells, mtCN recovered faster than TFAM levels, suggesting that mitochondrial biogenesis can occur without a proportional increase in TFAM expression [123]. In developing muscle cells, mtCN was decreased, despite a fourfold increase in TFAM expression [122]. Therefore, increased TFAM expression does not necessarily drive increased mitochondrial biogenesis. In a tissue-specific POLRMT KO, mtCN was decreased, despite normal TFAM levels [79], suggesting that, in the absence of POLRMT, TFAM-driven mitochondrial biogenesis is compromised. 

It has been proposed that TFAM is a general repressor of mtDNA expression, and this effect can be counterbalanced by tissue-specific expression of regulatory factors [100]. This view contradicts the abovementioned designation of TFAM as the master regulator of mitochondrial biogenesis. Collectively, the available evidence rules out the use of TFAM as a marker of mitochondrial biogenesis.

## 13. TFAM Orthologs

It has been suggested that sequence-specific DNA binding upstream of mitochondrial promoters is critical for TFAM-mediated transcription initiation and, indeed, mtDNA replication, considering that mitochondrial transcription generates primers for mtDNA replication at O_H_ in the classical strand-displacement model for mtDNA replication [143]. The notion that mitochondrial transcription (and TFAM as the key transcription factor) is essential for mtDNA replication is supported by the observation that KO of POLRMT or TFB2M, the other two critical players in mitochondrial transcription, results in the loss of mtDNA [25]. In its simplest form, an extension of this reasoning suggests that sequence-specific mtDNA binding by TFAM may be critical for mtDNA replication.

Both mtDNA and TFAM sequences vary, both between and within taxa, and it appears plausible that variations in the TFAM sequence may represent adaptations to changes in the mtDNA sequence. Looking at oTFAMs, they must all conserve the ability to bind DNA nonspecifically and yet be able to recognize specific DNA sequences, which are distinct between species. The difference in sequence-specific constraints should be reflected in TFAM sequence differences. From this perspective, examination of the ability of oTFAMs to support replication and transcription of h-mtDNA may be instructive. Recently, a newly developed GeneSwap approach was applied to examine the ability of oTFAMs to replicate h-mtDNA [25]. Since mtDNA promoter sequences and TSSs have not been mapped in most species whose TFAMs were examined in that study, the study generated no insights into species-specific TFAM–mtDNA interactions. However, it established that neither phylogenetic closeness nor sequence similarity determines the ability of oTFAM to support h-mtDNA replication. Indeed, oTFAM from the frog *Xenopus laevis* (37% aa identity) supported h-mtDNA replication, whereas oTFAM from the mammal opossum *Monodelphis domestica* (48.7% aa identity) did not [25]. Instead, it appears that the ability of oTFAMs to support h-mtDNA replication is driven by the variances in aa that make DNA contact, rather than by overall aa similarity [118].

## 14. Limitations of the Available Experimental Systems

One of the current obstacles to better understanding the role of TFAM in mitochondrial biology is the limitations of the available experimental approaches. In vitro systems afford unprecedented control of experimental conditions; however, they are reductionist and may not contain all the components available in vivo to support a given process with TFAM involvement. As a result, seemingly minor alterations in experimental conditions may result in opposite outcomes. Above, we already discussed the reversal of the orientation of TFAM binding in TFAM:HSP1 promoter crystals upon the addition of POLRMT and TFB2M and partial DNA melting in [50] vs. [42], the reversal of the LSP transcription sensitivity to TFAM C-terminal truncations upon extension of the transcription template in [34,35,60] vs. [54], and either the reduction of TFAM’s affinity to DNA [56] vs. no change in affinity [58] vs. an increase in affinity [57]. Therefore, in vitro data, while extremely useful for model generation, require validation in vivo or in situ. 

In vivo (in animals) experiments are the gold standard in biological research. Yet, these experiments are time-, labor-, and resource-expensive and are, therefore, inherently low-throughput. In addition, these experiments are not as tractable as those in vitro or in situ (in cultured cells). For example, mice with Ckmm promoter-driven TFAM KO specific to the heart and skeletal muscle survived for 2–4 weeks in [120], for 12 weeks in [28], and up to 21 week (150 days) in [31]. 

Inactivation of the genes that play key roles in mtDNA transcription and replication, such as TFAM, is embryonically lethal. This necessitates conducting animal studies using tissue-specific KOs. This mode of experimentation has two important limitations: First, each organ is composed of parenchymal cells that perform tissue-specific functions (e.g., cardiac myocytes in the heart) and stromal (supporting) cells (e.g., fibroblasts, endothelial or smooth muscle cells in the heart). Inactivation of the gene of interest in the parenchymal cells using tissue-specific-promoter-driven expression of a recombinase typically does not result in this gene’s inactivation in stromal cells. Yet, most biochemical assays are performed in whole-organ lysates composed of the contents of both parenchymal and stromal cells. Depending on the tissue, the parenchymal/stromal cell ratio can vary. For example, in the heart, cardiac myocytes constitute 25% by number and 75% by volume [144,145]. This makes it difficult to precisely evaluate the input of each cell type in an assay, and this is related to the second limitation of in vivo systems, which is that recombinases rarely recombine 100% of available targets, even under optimal conditions. Moreover, expression of a tissue-specific-promoter-driven recombinase does not guarantee recombination in every cell. Therefore, tissue-specific KOs are frequently mosaics [146,147]. Finally, some “tissue-specific” promoters are active in more than one tissue. For example, the Ckmm promoter is active predominantly in the heart and skeletal muscle, but also in the diaphragm and esophagus [148]. Therefore, at least in theory, the death of tissue-specific TFAM KO driven by the Ckmm promoter can be attributed to insufficiency of either cardiac or respiratory (skeletal) muscle.

In situ systems are intermediate between in vitro and in vivo systems in terms of throughput, tractability, cost, and limitations. Recently, a GeneSwap approach that enables in situ reverse genetic analysis of proteins involved in mtDNA replication and transcription has been reported [25]. This method is devoid of many limitations of in vitro, in vivo, and previous in situ systems. This approach provided new lines of evidence in support of the three-promoter model of mitochondrial transcription, the essentiality of TFAM for mtDNA replication, and the absence of essential nuclear function(s) of TFAM. It also enabled the exploration of TFAM permissible sequence space and identification of the leading role of HMG2 in TFAM sequence specificity, as well as helping to identify residues making mtDNA contact as the leading determinants of mtDNA replication; established the dispensability of the TFAM C-terminal tail for mtDNA transcription and replication; and demonstrated the genetic separability of TFAM’s contributions to mtDNA transcription and replication, etc. [25,43,118].

In general, the future progress of TFAM research strongly depends on improvement of the existing experimental approaches and the development of new ones.

## 15. TFAM in Disease

In total, 181 variants in the TFAM gene are listed in the UniProt database, of which 4 are predicted to result in premature termination of the polypeptide chain. However, only two pathogenic TFAM variants have been described in human patients. A c.533C > T (p.Pro178Leu) mutation was reported in a consanguineous kindred of Colombian–Basque descent. Two siblings presented with severe intrauterine growth restriction, elevated transaminases, conjugated hyperbilirubinemia, and hypoglycemia. Both patients died of progressive liver failure in early infancy [149]. TFAM protein expression in the patients’ fibroblasts was reduced, while TFAM mRNA was increased, suggesting that mutation may have affected TFAM mRNA translation or protein stability. Curiously, Pro178 may contribute to the HMG2 wedge into mtDNA [36,50]. The mtCN in fibroblasts was reduced to 40% of its normal value, which is still within 40–150% of the clinically normal range [150]. Pro178 is not conserved in TFAM orthologs that support h-mtDNA replication [25], suggesting that the reduced mtCN is not likely due to defective mtDNA replication. Subsequently, Mehmedovic et al. established that this TFAM variant is deficient in transcription initiation [58].

Another study described three affected individuals from a consanguineous family affected by variable seizures and intellectual disability, which segregated with c.694C > T, p.Arg232Cys mutation in TFAM [151]. Additionally, females developed primary ovary insufficiency, while the male had abnormal sex hormone levels. At the molecular level, TFAM protein levels were unaffected, but mtCN was modestly reduced, while remaining within the normal range. The number of mitochondrial nucleoids decreased, but their size increased [151]. Previously, this TFAM variant was shown to be defective in DNA binding and transcription activation [34]. Further, this aa is conserved among oTFAMs [25].

Given the critical role played by TFAM in cellular physiology through its contributions to mtDNA maintenance and expression, it is somewhat surprising that only a few pathogenic mutations have been described in this protein. However, this observation is in good agreement with a recent study that examined the functionality of oTFAMs in human cells and identified 730 substitutions conditionally permissive for mtDNA replication [25].

## 16. Concluding Remarks

Thirty-five years of TFAM research has dramatically improved our understanding of this protein’s structure and function. However, much remains to be learned. Resolving outstanding questions would facilitate progress in the field. Some of these questions are:

1. What are TFAM’s contributions to mitochondrial transcription beyond the LSP and HSP1? Current models of mitochondrial transcription limit their scope to these two promoters.
2. What are the structures of mitochondrial transcription complexes in situ? In vitro studies have suggested that POLRMT and TFB2M and/or partial DNA melting can reverse the orientation of TFAM binding at HSP1. Could other proteins further modify the structure of transcription complexes in situ (e.g., reverse the orientation back)?
3. How should the TFAM residues that make DNA contact be defined, and what are the identities of those residues? The evidence from TFAM:LSP crystals suggests that some residues (e.g., Leu182) may either intercalate into DNA [33] or make no contact with DNA [36,50].
4. What are the determinants of TFAM sequence-specific DNA binding?
5. If sequence-specific TFAM binding upstream of the mitochondrial promoter is an initiating event in the assembly of the transcription complexes, how does the orientation of TFAM binding upstream of the HSP1 promoter invert upon the transition to open complex? Is TFAM dissociation from DNA involved in the process?
6. To what extent does the statement that “TFAM ‘coats’ mtDNA” reflect the actual situation in situ?
7. What are the mechanistic basis and biological significance of observed uneven TFAM distribution between nucleoids within the same cell?
8. What is the mechanism of the resistance of free (not DNA-bound) TFAM to degradation by proteases in POLRMT KO cells [79]?
9. What mechanisms govern mtDNA persistence and transcription in tissues of tissue-specific TFAM KO animals?

We believe that further progress will be driven, in large part, by the development of new experimental approaches and the improvement of existing ones.

## Figures and Tables

**Table 1:** Variability in the annotation of h-TFAM domain boundaries.

h-TFAM Domains Expanse/Length (aa)						Reference
MTS	Leader	HMG1	Linker	HMG2	Tail	
1–42/42	-	43–121/79	122–151/30	152–221/70	222–246/25	[37]
1–42/42	-	43–125/83	127–153/27 *	154–221/68	222–246/25	[34]
1–42/42	-	43–122/80 **	122–152/31 **	152–222/71 **	222–246/25 **	[36,42,44]
1–49/49	-	50–118/69	119–154/36	155–219/65	220/246/27	[32]
1–42/42	1	44–120/77	124(123)–152/29–30 ***	153–225/73	226–246/21	[33]
1–42/42	7	50–122/73	123–152/30	153–223/71	224–246/23	[45]
1–42/42	7	50–115/66	116–154/39	155–234/80	235–246/12	[46,47]
1–42/42	7	50–118/69	119–154/36	155–219/65	220–246/27	[25,41]

* There is inconsistency in the delineation of domain boundaries (unassigned aa). ** aa overlap between domains. *** Different annotations in the text and Figure 1.

**Table 2:** TFAM residues in contact with DNA *.

	LSP [29]	LSP [32]	LSP [42] **	HSP1 [46]	HSP1 [42] **	Nonspecific DNA [46]
HMG1		Lys51	Lys51		Lys51	
		Lys52	Lys52	Lys52		Lys52
		Ser55			Ser55	Ser55
		Ser56			Ser56	
	Tyr57	Tyr57	Tyr57	Tyr57	Tyr57	Tyr57
	Leu58	Leu58	Leu58	Leu58	Leu58	
	Ser61	Ser61				Ser61
				Leu65		Leu65
	Lys69					Lys69
	Thr77	Thr77		Thr77	Thr77	
	Thr78	Thr78	Thr78	Thr78		Thr78
	Ile81	Ile81		Ile81		Ile81
	Arg82	Arg82	Arg82			Arg82
	Trp88	Trp88			Trp88	Trp88
	Arg89	Arg89		Arg89		Arg89
		Gln100	Gln100		Gln100	Gln100
		Tyr103	Tyr103	Tyr103	Tyr103	
			Trp107		Trp107	
Linker	Lys136					
		His137				His137
	Lys139					Lys139
	Arg140	Arg140		Arg140	Arg140	Arg140
	Met143					Met143
	Lys145					
	Lys146		Lys146	Lys146		
		Lys147	Lys147		Lys147	
HMG2	Thr150	Thr150				
	Lys156		Lys156		Lys156	Lys156
	Arg157	Arg157	Arg157	Arg157	Arg157	Arg157
	Arg159	Arg159	Arg159		Arg159	Arg159
	Tyr162	Tyr162	Tyr162	Tyr162	Tyr162	Tyr162
	Asn163	Asn163	Asn163	Asn163	Asn163	Asn163
	Val166					
			Ala167	Ala167	Ala167	
					Phe170	
	Pro178	Pro178		Pro178		Pro178
	Gln179	Gln179	Gln179	Gln179	Gln179	
	Leu182	***	Leu182	***		**
				Lys186		Lys186
C-Ter	Trp189	Trp189		Trp189		Trp189
			Glu208		Glu208	Glu208
	Tyr211	Tyr211				
	Arg232	Arg232	Arg232			
	Arg233	Arg233	Arg233		Arg233	
	Thr234	Thr234				

* Based on Figure 2 in [33] and Supplementary Figure 2 in [50]. ** The data were derived by running NucPlot on 6ERP and 6ERQ [42]. *** Even though L182 is not listed as intercalating residue in Ref. [50] Supplementary Figure 2, L182 is identified as intercalating residue of HMG2 in the main text.

## Data Availability

The data presented in this study are contained in the article.
